# Tattoo-associated *Mycobacterium haemophilum* Skin Infection in Immunocompetent Adult, 2009

**DOI:** 10.3201/eid1709.102011

**Published:** 2011-09

**Authors:** Meagan K. Kay, Tara R. Perti, Jeffrey S. Duchin

**Affiliations:** Author affiliations: Centers for Disease Control and Prevention, Atlanta, Georgia, USA (M.K. Kay);; Public Health–Seattle and King County, Seattle, Washington, USA (M.K. Kay, J.S. Duchin);; HealthPoint, Renton, Washington, USA (T.R. Perti);; University of Washington, Seattle (J.S. Duchin)

**Keywords:** Mycobacterium haemophilum, tattoo, skin infection, immunocompetent, tuberculosis and other mycobacteria, dispatch

## Abstract

After a laboratory-confirmed case of *Mycobacterium haemophilum* skin infection in a recently tattooed immunocompetent adult was reported, we investigated to identify the infection source and additional cases. We found 1 laboratory-confirmed and 1 suspected case among immunocompetent adults who had been tattooed at the same parlor.

*Mycobacterium haemophilum*, a nontuberculous mycobacterial species, typically affects immunocompromised persons. It produces subcutaneous nodules, papules, and pustules; less commonly it produces septic arthritis, osteomyelitis, pneumonitis, and disseminated infection (*1**,**2*). This organism causes lymphadenitis in healthy children (*3*) but rarely affects immunocompetent adults (*4*). Although other species of nontuberculous mycobacteria, predominantly rapidly growing species, have been associated with wound infections, cosmetic surgery, body piercing, and tattooing (*5**–**7*), *M.* *haemophilum* infection rarely has been reported as a complication of tattooing (*8**,**9*).

In November 2009, Public Health–Seattle and King County was notified of a chronic skin infection in an immunocompetent adult who had been recently tattooed; *M. haemophilum* had been isolated from the patient’s skin lesions. We investigated to characterize the clinical features of the case, determine the source of the infection, and identify additional cases.

## The Study

In August 2009, a healthy 44-year-old man (patient 1) received a tattoo on his left forearm at a commercial tattoo parlor. Three days later, a painless rash developed at the tattoo site. He applied antibacterial ointment, but the rash did not resolve; 12 days after rash onset, he sought care from his health care provider. The patient denied fever and other focal or constitutional symptoms. Erythematous nodules of 3–5 mm diameter in the region of the tattoo were noted, and the patient was given ceftriaxone and trimethoprim/sulfamethoxazole for presumed pyogenic infection. Two weeks later, the lesions were unimproved. Aerobic culture of the lesions was conducted and clindamycin was prescribed; no organisms grew from the culture. In mid-September, the patient again visited his health care provider because the nodules remained unimproved. Ceftriaxone was administered, and oral cephalexin was prescribed; an aerobic bacterial culture was repeated. Two weeks later, the numerous nodular pustules confined to the tattoo region remained (Figure).

**Figure:**
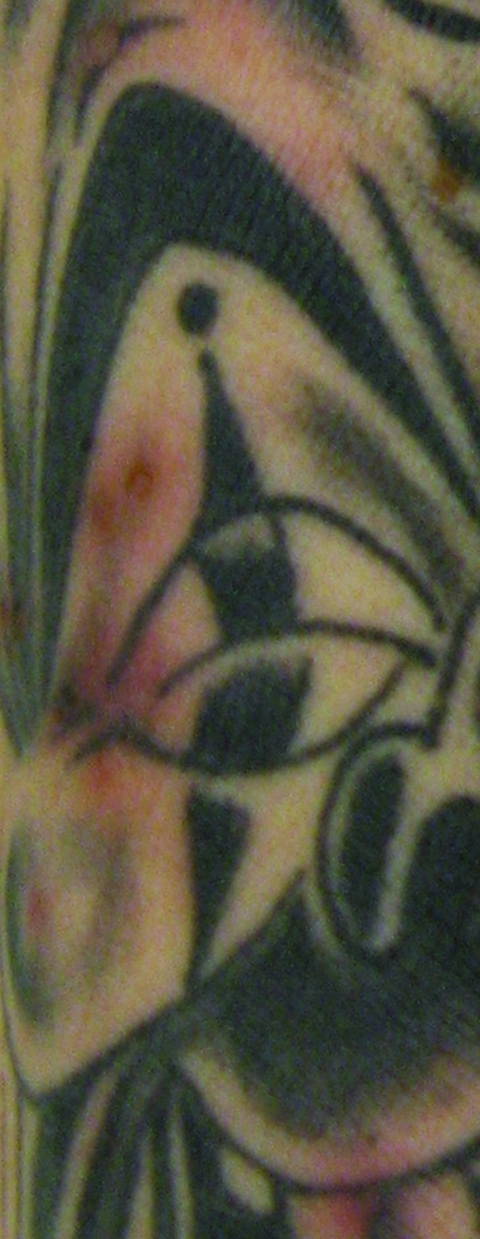
Pustular rash caused by *Mycobacterium haemophilum* confined to the tattooed region of the forearm. Photograph taken in October 2009, two months after tattooing.

Test results for hepatitis B and C viruses and HIV were negative. A swab of purulent material from 2 pustules was submitted for aerobic bacterial and fungal culture, an acid-fast bacilli (AFB) culture and smear, and a varicella-zoster virus direct fluorescent antibody assay and culture; clindamycin was prescribed. Samples were spread onto Middlebrook and chocolate agar plates and incubated at 30°C and onto Middlebrook agar plates and incubated at 37°C. After 3 weeks, AFB were recovered from only the plates incubated at 30°C. Using 16S rRNA gene sequencing, we identified the isolates as *M. haemophilum.* The organism was sensitive to clarithromycin (<15 µg/mL), rifampin (<1 µg/mL), trimethoprim/sulfamethoxazole (<0.5/9.5 µg/mL), amikacin (<12 µg/mL), linezolid (<6 µg/mL), ciprofloxacin (<2 µg/mL), and moxifloxacin (<5 µg/mL) (*10*).

In December 2009, treatment with rifampin, ciprofloxacin, and clarithromycin was initiated. In February 2010, the rash had improved, although healing papules and erythema were still present. In March 2010, the patient discontinued treatment because of nausea. By May 2010, the lesions had healed.

In mid-October 2009, the same health care provider evaluated a healthy 35-year-old man (patient 2) with a pustulo-nodular skin infection confined to shaded areas in a tattoo received in August 2009 at the same tattoo parlor. During November–December 2009, standard aerobic bacterial or mycobacterial cultures from this patient’s lesions were performed, but no organisms were recovered. We considered this to be a suspected case.

During December 2009, both patients were interviewed; no other potential epidemiologic links were identified. Each patient denied exposure to recreational water, aquarium water, water with rusty sediment, or any other potential skin irritants.

To identify additional *M. haemophilum* cases, Public Health–Seattle and King County asked physicians to report atypical skin infections that developed after receipt of tattoos performed during June 1–December 1, 2009, and asked clinical laboratories to report atypical mycobacterial species recovered during the same period. No additional cases were identified.

During an investigation of the tattoo parlor on December 10, 2009, the operator reported having used similar procedures to tattoo each patient. No deviations from Washington State safety and sanitation standards were recognized (*11*). Municipal water was used in a rinse solution applied during and after tattooing and to dilute ink for shading. Eleven environmental samples collected during the site visit included ink (1.5 L); tap water (1.5 L); liquid soap (1 L); petroleum jelly; and swabs of equipment, the soap dispenser port, and the tip of a reusable black-ink container. All samples were submitted to the Centers for Disease Control and Prevention (Atlanta, GA, USA) for mycobacterial culture; no mycobacteria were recovered. The tattoo parlor operator was instructed to use only sterile water for rinse solutions and dilution of tattoo dye.

## Conclusions

Although the infectious agent was confirmed by culture for patient 1 only, the infection for patient 2 was consistent with *M. haemophilum* infection and patient 2 was epidemiologically linked to patient 1. The nonspecific rash that developed 3 days after tattooing for patient 1 might be unrelated to *M. haemophilum*; however, the development of pustular nodules after 2 weeks is consistent with the incubation period for this infection. Although punch biopsies are typically required for diagnosis of nodular lesions, *M. haemophilum* was cultured from a swab of the lesions. The pustules were similar to those previously reported for tattoo-associated *M. haemophilum* infection (*8*) and might be associated with the presumed mode of inoculation.

Although the environmental reservoir for *M. haemophilum* is unknown, the organism is thought to be widespread in the environment (*2*). Water has been a suspected reservoir because of the epidemiology of other environmental mycobacteria and because *M. haemophilum* has been detected by PCR in biofilms from research aquariums (*12*). However, in most investigations, culture of *M. haemophilum* from environmental samples has been futile (*2**,**5*). The interval of >4 months between the time patient 1 was tattooed and the environmental sample was collected might have further reduced the likelihood of recovering *M. haemophilum*. Molecular methods such as PCR might be more successful than culture alone for detecting *M. haemophilum* infections.

No tattoo industry standards exist for the practice of diluting tattoo ink. Washington State does not specifically require tattoo artists to use steam-distilled or sterile water when rinsing needles or diluting ink; tap water is often used (*11*). However, legislation enacted in July 2010 prohibits mixing ink and pigments with improper ingredients (*11*). Although infections attributable to water appear uncommon, we advise against using tap water for tattoo procedures.

Treatment for *M. haemophilum* infection among immunocompetent adults should be based on that used for immunocompromised patients for whom multidrug regimens, including clarithromycin, rifampin, rifabutin, and ciprofloxacin, are recommended (*1**,**2**,**13*). Agents that seem to be active in vitro are amikacin, clarithromycin, ciprofloxacin, rifampin, and rifabutin (*1**,**14*). Isolates have variable susceptibility to doxycycline and sulfonamides and are typically resistant to ethambutol, isoniazid, and pyrazinamide (*1**,**13*). However, because no standardized methods for assessing antimicrobial drug susceptibility of *M. haemophilum* exist, in vitro susceptibility data must be used with caution.

Clinicians should consider *M. haemophilum* in the differential diagnosis of skin infections after tattooing, particularly chronic skin infections that are unresponsive to treatment with antimicrobial drugs, regardless of the patient’s immune status. *M. haemophilum* infections can be difficult to diagnose because the organism is slow growing and fastidious and requires iron supplementation and a lower incubation temperature for growth (30°–32°C) than other mycobacteria (*15*). Laboratory practices vary, and hemin might not be routinely added to all AFB cultures. Therefore, for suspected cases, clinicians should alert the laboratory to use appropriate procedures to culture for *M. haemophilum* and other AFB.
